# Critical Care Nurses’ Knowledge of Correct Line Types for Administration of Common Intravenous Medications: Assessment and Intervention Study

**DOI:** 10.2196/36710

**Published:** 2022-04-26

**Authors:** Rania Al-Jaber, Natalie Samuda, Ahmad Chaker, James Waterson

**Affiliations:** 1 Pharmaceutical Care Division King Faisal Specialist Hospital and Research Centre Riyadh Saudi Arabia; 2 Clinical Support Services Medication Management Solutions Becton Dickinson Dubai United Arab Emirates; 3 Pharmacy Services Cleveland Clinic Abu Dhabi United Arab Emirates; 4 Medical Affairs Medication Management Solutions Becton Dickinson Dubai United Arab Emirates

**Keywords:** critical care, intravenous medication, compatibility, administration error, infusion maintenance, medication interaction, knowledge, survey

## Abstract

**Background:**

There is a paucity of information in the literature on core nursing staff knowledge on the requirements of specific intravenous administration lines for medications regularly given in critical care. There is also a lack of well-researched and appropriate information in the literature for intravenous administration line selection, and the need for filtration, protection from light, and other line-material selection precautions for many critical and noncritical medications used in these settings to maintain their potency and efficacy.

**Objective:**

We aimed to assess the knowledge gap of clinicians with respect to intravenous administration line set material requirements for critical care medications.

**Methods:**

Data were drawn from a clinician knowledge questionnaire, a region-wide database of administered infusions, and regional data on standard and special intravenous administration line consumption for 1 year (2019-2020) from an enterprise resource planning system log. The clinician knowledge questionnaire was validated with 3 groups (n=35) and then released for a general survey of critical care nurses (n=72) by assessing response dispersal and interrater reliability (Cronbach α=.889). Correct answers were determined by referencing available literature, with consensus between the team’s pharmacists. Percentage deviations from correct answers (which had multiple possible selections) were calculated for control and test groups. We reviewed all 3 sources of information to identify the gap between required usage and real usage, and the impact of knowledge deficits on this disparity.

**Results:**

Percentage deviations from correct answers were substantial in the control groups and extensive in the test group for all medications tested (percentage deviation range –43% to 93%), with the exception of for total parenteral nutrition. Respondents scored poorly on questions about medications requiring light protection, and there was a difference of 2.75% between actual consumption of lines and expected consumption based on medication type requirement. Confusion over the requirements for low-sorbing lines, light protection of infusions, and the requirement for filtration of specific solutions was evident in all evidence sources. The consumption of low-sorbing lines (125,090/1,454,440, 8.60%) was larger than the regional data of medication usage data would suggest as being appropriate (15,063/592,392, 2.54%).

**Conclusions:**

There is no single source of truth for clinicians on the interactions of critical care intravenous medications and administration line materials, protection from light, and filtration. Nursing staff showed limited knowledge of these requirements. To reduce clinical variability in this area, it is desirable to have succinct easy-to-access information available for clinicians to make decisions on which administration line type to use for each medication. The study’s results will be used to formulate solutions for bedside delivery of accurate information on special intravenous line requirements for critical care medications.

## Introduction

### Background

There is a paucity of information in the literature on core nursing staff knowledge on the requirements of specific intravenous lines for medications regularly administered in settings such as critical care, coronary care, and high-dependency units. This is matched by the lack of well-researched and appropriate information in the literature on material compatibility, the need for filtration, protection from light, and other selection precautions for many medications administered intravenously in these settings.

Within critical care, and particularly in pediatric and neonatal critical care, evidence is beginning to emerge that incompatibility between medications can cause the precipitation of medications in the venous access device [1], which may result in intravenous access failure or partial occlusion of peripheral access devices and central lines. A recent study [2] showed that venous access occlusion alarms are responsible for 55% of all intravenous infusion pump alarms in neonatal intensive care units and that noninfusion of critical short–half-life infusions (due to such occlusions) has the potential to cause severe cardiovascular instability in critical care patients receiving vasoactive medications. Maintaining line patency and ensuring noninterruption of medications such as inotropes and cardiac glycosides are very much patient safety issues [3].

The problem of limited access and multiple infusions running into a single venous access device is common in critical care, particularly in pediatric and neonatal critical care settings. Fonzo-Christe et al [1] suggested that the filtration of medications might be one method of reducing the risk of precipitation during multiple concurrent infusions; however, the questions—for which medications? and how can we ensure that nursing staff are aware of and able to comply with such requirements?—should be asked.

Critical care costs are high and increasing. Pharmaceutical costs are a sizeable component of these costs. Indeed, in all hospital areas, the price of pharmaceuticals is increasing rapidly year-on-year with a 30.1% rise in per capita spending (adjusted for purchasing power parity) between 2010 and 2018 across Organization for Economic Cooperation and Development nations, despite overall consumption only increasing by 7.1% in the same period [4,5]. Cost reduction approaches undertaken by health care systems, however, often ignore the actual use of these medications once they are purchased; storage, compounding, and administration of these medications are areas in which value and efficacy can be lost and waste can be generated through incorrect medication management.

With some medications, the manufacturer’s instructions for administration are clear, but with others, the manufacturer’s instructions may give information on storage only, for example, Pfizer’s package insert for sodium nitroprusside has very clear statements about the requirement for light-protected administration postdilution [6]; however, the same active molecule marketed as a generic by another manufacturer does not discuss protection during administration [7].

Even if pharmaceutical manufacturers do give adequate guidance on storage and protection of base medications, there is generally much more sparse information available to the end-user postreconstitution or after compounding processes have been undertaken. Furthermore, package inserts will usually remain within the central pharmacy if compounding is undertaken in isolated intravenous clean rooms, as per many accreditation bodies and pharmaceutical safety societies’ recommendations for medication compounding and reconstitution [8], and therefore, may be unavailable for consultation by the nursing staff who administer the medication.

The diluent used for intravenous medication compounding and can also change the requirements for protection from light and filtration, for example, epinephrine (adrenaline) is photostable when mixed in normal saline, but it is photolabile when mixed in dextrose solutions [9]. Even well-constructed and researched bedside intravenous administration manuals, such as those of the Royal Children’s Hospital in Melbourne [10] and the British National Formulary [11], do not generally include information on material selection, guidance on protection from light, and required filtration of the compounded medication for intravenous administration lines.

Clearly, more could be done in terms of labeling medications requiring special administration lines during centralized compounding processes to give nurses better information on administration. Some intravenous smart pumps carry clinical advisories on their user interfaces, which are customizable for individual medications, and these have been successfully used in failure modes and effects analysis of intravenous medication safety strategies for reducing medication administration errors by users. The user cannot proceed with programming without confirming that they have read the advisory. In one study [12], a medication safety strategy that used centrally applied medication labels from the central compounding service to match the dose, dilution, advisories, and warnings as presented on the patient’s electronic medication administration record (aligned with the medication information available on the automated dispensing cabinet and integrated refrigeration unit and on the intravenous smart pump) was described. The drawback of this approach is that the nurse has often primed the intravenous administration line manually (either by gravity for a volume pump or by manual purge in the case of a syringe driver); therefore, the clinical advisory may be ignored in the interest of convenience, either because a considerable amount of pump programming has already taken place to get to the clinical advisory or in order to avoid wasting the medication that has already travelled through the intravenous administration set (this can be around 20 mL with most volume pump intravenous administration sets, thus, not an inconsiderable amount, particularly in the case of pediatric and neonatal patients).

A second issue is that despite the growth of central intravenous additive services in many facilities many critical care medications are still compounded by nurses at the bedside or in the medication room. Critical care prescriptions are not uncommonly urgent orders, and the consumption of medications can be so large and rapid that even a well-functioning central compounding unit may struggle to keep up with demand.

The above is also based on the assumption that connected and integrated intravenous smart pumps are available to update clinical advisories to match compounding changes. This scenario is rare. Many intravenous smart pumps are not connected to a central server, particularly when they are deployed to dispersed high-dependency beds or to infection-isolation units. Wireless connectivity can facilitate updates, but with older intravenous pumps, updates are dependent on biomedical engineers uploading new libraries manually to each intravenous smart pump [12,13].

A recent survey of sterile compounding errors indicated that 74% of survey respondents were aware of at least one pharmacy sterile compounding error in the preceding 12 months, for the purposes of the current study what was particularly pertinent in the survey was that 41% of these errors involved labelling of a compounded sterile preparation (including omission of administration guidance) and that wrong preparation techniques, including the attachment of an incorrect administration line, were found in 26% of compounding errors [14]. Approximately 30% of the degradation of clear medication solutions due to light occur in the line or tubing of the infusion set [9].

It is a common misconception that all intravenous administration lines are the same. They are not. Incorrect administration line selection by the clinician can cause deleterious changes to the active ingredients in the medication being given. Not only is this potentially harmful to the patient, as chemical change may take place in the medication upon contact and interaction with administration line material (for example, the creation of peroxides when total parental nutrition is exposed to light during administration), but there may also be a significant loss in the efficacy of the medication and degradation of its therapeutic value. Not only is this equivalent to incorrect administration of the medication, it is also potentially a waste of the full monetary value of the medication. There are a small number of papers delineating a significant financial impact to organizations from incomplete intravenous medication dosing and subsequent requirement for redosing [15,16].

Up to 80% of most facilities’ infusion activities take place in critical care. This is also an area where stat-prescribing is common, where first doses of many medications are commonly not prepared in pharmacy, and where compounding by nursing staff is also common.

### Objectives

The study had 4 goals. First, we wanted to create a verified, reliable, dependable, and easily replicated survey tool to assess the knowledge gap of clinicians in terms of their abilities to select the correct administration set for medications commonly encountered in their care area. Second, we wanted to investigate the gap between what clinicians should seek to apply, in terms of infusion protection through the use of special administration sets and what, in fact, takes place in terms of consumption of these lines across average uses of critical care medications with known requirements in terms of material, filters and light protection. Third, we wanted to be able to use this information to consider solutions in terms of information availability and education leading to consistent and accurate selection of special intravenous administration lines. Finally, we wanted to suggest a strategy to ensure that the medication administration line information given to end users is up-to-date, pertinent, evidence-based, and appropriate for their specific needs.

## Methods

### Study Design and Data

We collected data using a clinician knowledge questionnaire via web-based survey, direct email request, and in-person (at critical care conferences). To compare perceived or potential usage based on survey responses with real-world critical care usage, regional data on standard and special intravenous administration line consumables for a 1-year period (2019-2020) and the amount of critical care medications, for which special intravenous lines (light-protective, filtered, or low-sorbing) were necessary, were obtained from two sources. We used an enterprise resource planning system log for the Middle East and North Africa. This database comprises 2.2 million infusions given across Europe and the Middle East, which has been previously used in a study on infusion pump alarms [2]. In addition, we reviewed individual hospital accounts.

### Clinician Knowledge Assessment Survey

#### Inclusion and Exclusion Criteria

Nurses working in critical care areas (adult intensive care units, pediatric intensive care units, neonatal intensive care units, high-acuity high-dependency units, and coronary care units) where parental drug administration of the medications addressed in the survey are commonly used were included.

Nursing staff working outside of critical care settings, with the exception of nurse educators and clinical leads with crossover roles within clusters of care areas (applied generally to critical care directorates), were not included.

#### First Pilot

We prospectively validated the clinician knowledge assessment survey. Prospective validation is widely used in such areas as the production of new and innovative practice guidelines, policies, and clinical decision-making tools to assess them for dependability [17].

The questionnaire was pretested among a convenience sample of clinicians, who were divided into 2 groups (A and B), with moderate to extensive experience with infusion therapy. Clinicians received an email invitation to take part in the survey. Reminder invitations were sent out at 3-day intervals to improve response rates. Our aim was to assess the clarity of wording and to verify the coherence of the questions. The groups were drawn from European, west Asian, and African countries where English was a second language. We evaluated answer groupings from each group and clustering or dispersal of answers. Overall, response dispersal for the survey (Cronbach α=.889) indicated interrater consistency (Cronbach α>.7 is acceptable).

Some minor changes to question language were made at this point in response to differences in answer clustering that may have been related to poor understanding of individual questions. For example, the nomenclature *epinephrine* was added to the survey tool with the original *adrenaline*. Overall, the questionnaire was deemed to be valid because the groups showed similar selection grouping and consistency in their answers. We discussed changing the phrase *light-protective* to *light-sensitive* because a lot of regional educational and guidance materials for nurses use the phrase *light-sensitive* to describe this special type administration line, but for clarity, we rejected the change—although medications may be described as *light-sensitive*, most authoritative texts use *light-protective* to describe administration lines [18]. We also discussed removing the question pertaining to the medication *epoprostenol*, because several respondents had commented that they were not familiar with this medication. We decided to retain the question based on its use in extracorporeal circuit management in many critical care units and its use in neonatal intensive care.

The response rate was 100%; we believe this was due to the succinct nature of the assessment, because respondents could complete it quickly.

#### Second Pilot

An introduction was added, to give respondents an impetus for completing the survey and to act as informed consent, and the questionnaire was released (Multimedia Appendix 1) in 20 countries via Google Forms, as a second-stage pilot. A total of 15 responses (Group C) were obtained. No further changes were made because good internal consistency was demonstrated and there was no evidence of spurious answers; the pattern of answers was similar to those in the initial pilot (Table 1). Though the number of respondents was small, this group was a useful adjunct because they, like the initial test groups, potentially had access to medication information from the internet. Although they were asked to complete the survey using their own knowledge, there was an opportunity for cheating. Groups A, B, and C formed the control group (n=25).

**Table 1:** Response dispersal during questionnaire validation.

Question	Medications assessed, n	Group A (n=10)	Group B (n=10)	Group C (n=15)
1	10	Dispersed over 5 choices	Dispersed over 6 choices	Dispersed over 5 choices
2	10	Dispersed over 5 choices	Dispersed over 5 choices	Dispersed over 5 choices
3	4	Concentrated over 2 choices	Mildly dispersed over 3 choices	Concentrated over 2 choices
4	4	Concentrated over 2 choices	Concentrated over 2 choices	Concentrated over 2 choices
5	4	Mildly dispersed over 3 choices	Dispersed over 4 choices	Dispersed over 4 choices
6	4	Mildly dispersed over 3 choices	Dispersed over 4 choices	Dispersed over 4 choices

#### Test

A test group of respondents (n=72) completed the survey independently. No access to internet information sources was permitted, and respondents were asked to complete the survey independently. The surveys were conducted in 2021 at the World Federation of Critical Care Nurses Congress and the Emirates Critical Care Congress, which were held in Dubai. Analysis of survey results for interrater reliability was undertaken using Minitab (version 18).

In assessing respondents’ answers as either correct or incorrect, we defined standard lines as those not made with di(2-ethylhexyl) phthalate, because infusion therapy is moving toward not using the plasticizer *di(2-ethylhexyl) phthalate* due to concerns over its interaction with multiple medications and fluids in even, what may be considered to be, nonspecial infusions [18] and special lines (Table 2), in accordance with definitions used by the other data sources.

Correct answers were defined based on the findings of an extensive literature search (Multimedia Appendix 2), and consensus between the team’s pharmacists (RAJ and AC) was required.

Answers from the control and test groups were calculated as percentage deviations from a fully correct selection. This approach was taken due to the nature of the questions, wherein multiple item selections could be made, leading to the chance of multiple wrong and correct selections. The selection of all medications correctly was scored as 0; an incorrect selection was scored –1, and the omission of a correct selection was scored as +1.

**Table 2:** Definitions and characteristics of standard and special intravenous administration lines [19-22].

Administration line type	Definition	Characteristics	Comments or usage example
Nonspecial line	Standard	Not made with di(2-ethylhexyl) phthalate Made with polyvinylchloride	General medication administration
Low-protein binding 0.2-micron filter	Specialty	Not made with di(2-ethylhexyl) phthalate Made with polyvinylchloride Polyurethane filter	Amiodarone, total parental nutrition (lipid-free, crystalloid, vamin)
Low-protein binding 1.2-micron filter	Specialty	Not made with di(2-ethylhexyl) phthalate Made with polyvinylchloride Polyurethane filter	Total parenteral nutrition containing lipid emulsion or lipid infusions
Low-sorbing line	Specialty	Not made with di(2-ethylhexyl) phthalate Made with polyvinylchloride Polyethylene lined	Insulin, nitroglycerin, alemtuzumab, bleomycin, cabazitaxel, docetaxel, ifosfamide
Low-sorbing line with low-protein binding 0.2-micron filter	Specialty	Not made with di(2-ethylhexyl) phthalate Made with polyvinylchloride Polyethylene lined Polyurethane filter	Epoprostenol, alemtuzumab, thiotepa, ofatumumab, panitumumab, cetuximab, paclitaxel
Light-protective	Specialty	Amber Not made with di(2-ethylhexyl) phthalate Made with polyvinylchloride	Adrenaline (epinephrine), amiodarone, labetalol, digoxin, bleomycin, doxorubicin, methotrexate, rituximab, vincristine

### Analysis of Intravenous Line Usage and Medication Usage Data

Regional data on standard and special intravenous line consumable usage were grouped by specific characteristics. The amount of medication for which special intravenous administration lines would be required was assayed using a software program (Alaris CQI, version 4.3; Becton, Dickinson and Company).

Mapping between these 2 sources of information was undertaken for evidence of the degree of discrepancy between actual use of special intravenous administration lines in the region and what should have been used assuming full knowledge of the intravenous administration line requirements for the total number of medications requiring special intravenous administration lines given. We suggest that any knowledge deficit identified in the survey of nurses could be an important cause for incorrect use (under or overuse) of special intravenous administration lines identified in the database contrasting.

## Results

It became evident that the test group found question 3 to be problematic, despite its interrater consistency; the interrater consistency was acceptable for all other questions (Table 3). Many respondents gave rapid feedback that the question was confusing, because they were confident that none of the medications required filtering. Respondents therefore appeared to have randomly selected 1 incorrect option. The question was, therefore, removed from final survey results.

There was a substantial discrepancy between actual usage (enterprise resource planning data on standard and special intravenous administration line consumable use) and correct usage of medication with special line requirements (Table 4).

**Table 3:** Interrater consistency (control and test groups).

Question	Item-adjusted total correlation	Squared multiple correlation	Cronbach α
1	0.2299	0.4336	.7923
2	0.7281	0.7493	.6978
3	0.1693	0.5101	.7966
4	0.6414	0.5419	.7117
5	0.6508	0.7266	.7045
6	0.7678	0.6426	.6636

**Table 4:** Enterprise resource planning and facility consumption and intravenous medication administration requiring special lines.

Specialty line type	Intravenous consumables usage (n=1,454,440), n (%)	Required usage based on medications administered (n=592,392), n (%)
Low-protein binding 0.2-micron filter	0 (0.00)	27,428 (4.63)
Low-protein binding 1.2-micron filter	6600 (0.45)	2987 (0.50)
Low-sorbing line	125,090 (8.60)	15,063 (2.54)
Low-sorbing line with low-protein binding 0.2-micron filter	6000 (0.41)	3407 (0.58)
Light-protective	109,400 (7.52)	60,812 (10.27)

## Discussion

### Principal Findings

A good example of the absolute need for the correct selection of administration line for a particular medication is that of epoprostenol. This medication requires light protection and 0.2-micron filtration and reacts with polyethylene (which is commonly used in low-sorbing lines). Question 6, which was related to this medication was poorly answered by respondents in the control group (percentage deviation range –19% to 41%) and answered particularly poorly in the test group (percentage deviation range –43% to 93%). Given its extremely short half-life and use of this medication for critical interventions such as extracorporeal circuit management and pulmonary hypertension treatment, degradation of the active molecule due to incorrect line selection has potentially serious effects.

Question 1, which was about medications requiring protection from light, also showed a large deviation in the test group (percentage deviation range –25% to 92%), with multiple omissions including short half-life vasopressors and cardiac medications. The control group (percentage deviation range –13% to 47%) performed far better on this question. Each group was asked to not use internet resources while answering each question, but individuals in the control group were unsupervised during the completion of the survey and may have submitted to the temptation to use medication guides on the internet.

**Figure 1:**
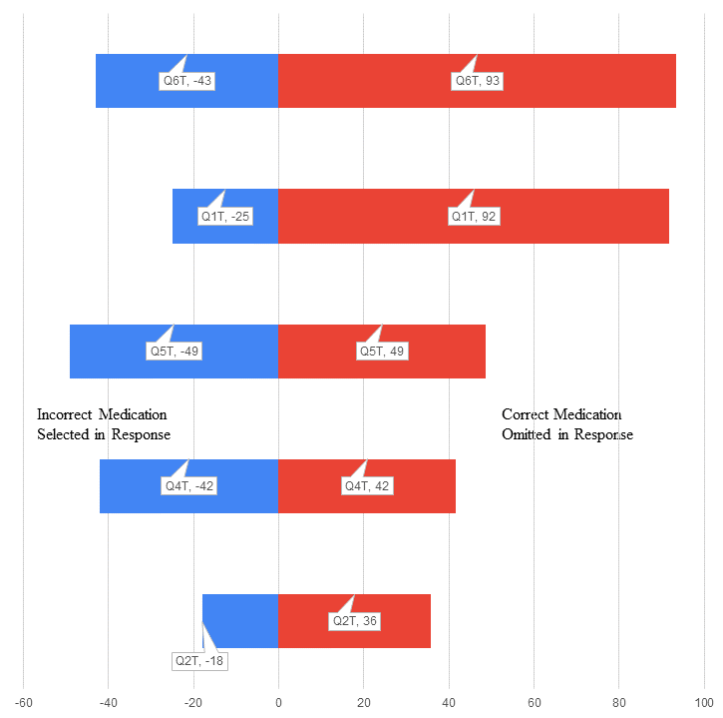
Tornado distribution plot (percentage deviation from correct selections) for the test group.

**Figure 2:**
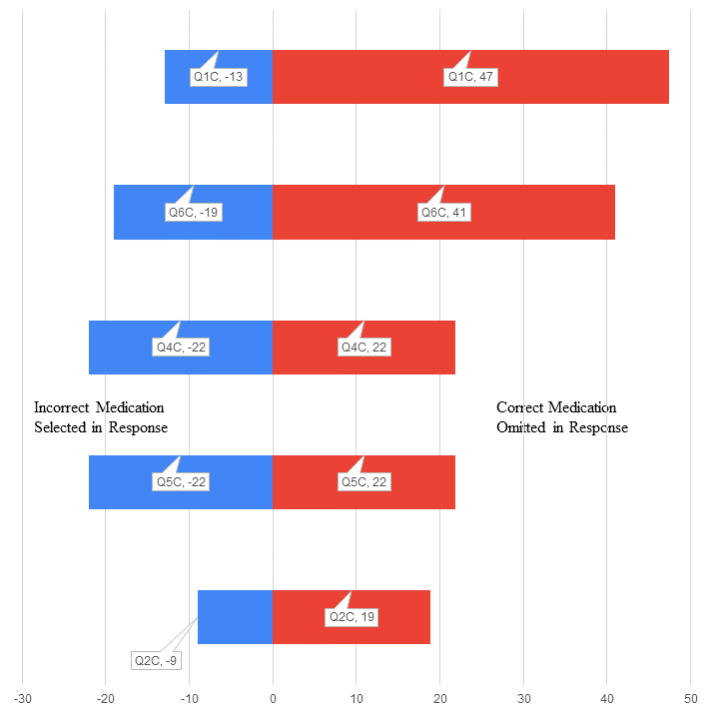
Tornado distribution plot (percentage deviation from correct selections) for the control group.

**Figure 3:**
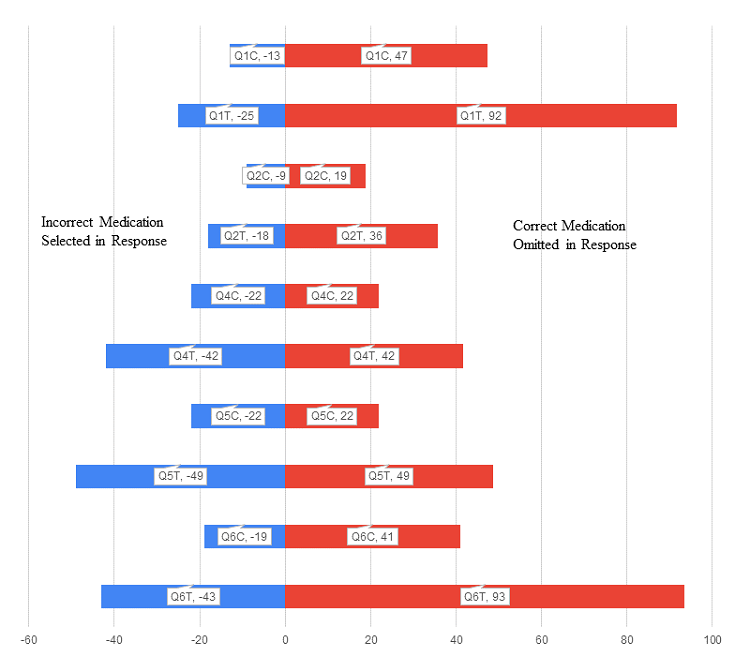
Distribution plots (percentage deviation from correct selections) for control and test groups.

In fact, the test group (Figure 1) consistently performed worse than the control group (Figure 2) for every question (Figure 3). There was no time limit applied to completion of the questionnaire for either group, but the opportunity to review medication guides on the internet or medication manufacturers’ package inserts cannot be discounted, and in the study, demonstrates that, although access to information improves performance, there is conflicting information and a lack of hierarchy associated with information on this topic. Reducing clinical variability has been a goal in health care for a considerable amount of time. It is desirable that we verify, concentrate, and distill information for clinicians.

There was a discrepancy between the relative amount of intravenous line consumables that should be used and that being consumed. This was notably evident for consumed (109,400/1,454,440) and required (60,812/592,392) light-protective lines—a difference of 2.75%.

There was reasonable agreement between usage percentages for the low-sorbing line combined with a low-protein binding 0.2-micron filter (consumed: 6000/1,454,440, 0.41%; appropriate: 3407/592,392, 0.58%) and the standard line with a low-protein binding 1.2-micron filter (consumed: 6600/1,454,440, 0.45%; appropriate: 2987/592,392, 0.50%). This may be related to the fact that these are products commonly used for total parental nutrition, either with or without lipids, and that instructions are usually marked very clearly on these products as they are almost exclusively dispensed (and very commonly compounded) by the pharmacy department. It is notable that, in the survey, both the control group (percentage deviation range –22% to 22%) and the test group (percentage deviation range –44% to 44%) showed substantially less deviation for the question on the filtration requirements for total parental nutrition containing lipids than those for the other questions.

Consumption of low-sorbing lines was substantially larger (125,090/1,454,440, 8.60%) than that indicated to be appropriate (15,063/592,392, 2.54%), which may be related to the fact that many facilities in the region use this particular line type for vasoactive infusion administration because the line itself has no lower Y-site, which helps mitigate against inadvertent flushes or push medications being administered into a line that is delivering medications that must be given at a constant and uninterrupted rate.

### Study Limitations

The study was limited to the Middle East for the main part of the survey, but the control group was composed of clinicians from Europe, the Middle East and Africa. There was certainly a small degree of discussion between some respondents at the in-person survey despite surveyors’ requests to complete the survey individually, but this behavior, in fact, imitates nurses’ decision-making practices [23].

The test group was predominately composed of nurses who work in intensive care units (all: 44/72, 61.11%; pediatric 14/72, 19.42%; neonatal: 12/72, 16.66%; high-acuity high-dependency: 2/72, 2.81%).

### Conclusions

The survey and the review of real-world data and enterprise resource planning data indicated a large degree of clinical variation.

The survey included medications that are in common use throughout critical care, and there was a large difference of opinion among respondents on line type and material required for their administration. This is worrisome, particularly because the medications in the survey are core intensive care unit medications—the risk of incorrect intravenous administration line selection would be expected to increase with unfamiliar medications. The competency pyramid is built on knowledge, skills, and attitude [24]. Competency is difficult to maintain with limited exposure to tasks such as reconstituting and administering uncommonly used medications, and the knowledge environment in which the clinician is operating may also be limited, with no experienced peers or easily accessible information available. In the last two years, we have observed multiple cases of staff working outside of their regular units to meet the needs of expanded critical care units, and crisis conditions that have largely precluded the full supply of central intravenous additive services medications to critical care units.

Furthermore, in critical care, the likelihood of an ideal situation for integrated compounding-administration, with products and information flowing from the central pharmacy to the bedside clinician who receives accurate information from the medication’s barcode label, including patient details, drug dose and volume, and important instructions for administration such as which intravenous administration line is required for safe, effective, and uncorrupted administration, and the product being tracked through connected inventory systems to ensure correct storage of the reconstituted product (particularly those requiring temperature control and protection from light) is unlikely [25]. The Institute for Safe Medication Practices [25] recorded that 28% of respondents to their survey in November 2020 reported “often or always admixing intravenous continuous infusions or titrations, particularly insulin, vasopressors, or lifesaving drug infusions required during emergencies.” Much of this reconstitution and mixing takes place outside of the medication room and away from compounding information sources; at the bedside (37%), nursing station desks (28%), and bedside computer workstations (16%) [25].

It is of even greater concern that 53% of the respondents of that survey [25] disagreed or strongly disagreed that their organization requires practitioners who prepare sterile injectable medications and infusions to undergo formal training. Similarly, 49% of respondents disagreed or strongly disagreed that they have been formally trained for the task, and 32% reported no formal training or annual competencies for compounding and reconstitution of intravenous medications [25]. The likelihood of a knowledge gap, not just in terms of dose and dilution but also in terms of administration line requirements for medications, is evident.

The need for a trusted bedside guide for administration line usage and compatibilities is evident. We have known about the ability for handheld and bedside applications to deliver immediate information at the bedside for a considerable time [26]. We suggest that the issue of guidance for administration line selection would be best managed by a handheld device app that could be downloaded to either dedicated facility-owned handheld devices which may have other functions such as barcode reading or alarm routing, or to so-called bring-your-own-device mobile phones or tablets. Any medication and special intravenous administration line guide needs to be accurate, current, and complete. This means that it requires a valid process for both initial and ongoing review. We propose that the best way to manage such a process would be via an initial Delphi panel [27] as this technique is structured around independent work—meetings are kept to a minimum and the participants can be in distant locations and time-zones and still participate. It has also been used to address complex and difficult questions in health care where expert opinion is often the only method of obtaining a cogent outcome, for example, acceptable limits of polypharmacy and types of medication that can be safely prescribed for older adults [28,29]. This could be followed by annual review via the same method. (The initial panel should be tasked with setting this review period.) The findings of this panel could then be incorporated and presented in an easy-to-use guide.

We concentrated on critical care medications, but in the future, we would like to further extend the survey technique to a review of intravenous oncology medications, as this is an area where special administration lines are often required and where loss of efficacy or even debasing of the active molecule has serious consequences for patients and for the facility if cycles of chemotherapy require repeat administration because of incorrect administration [4].

One particular issue with all medication regimens, but one which has a particular resonance with high-value medications and for pediatric oncology, is ensuring a complete dose. Loss of efficacy is a risk, but running infusions to completely empty the intravenous tube of all medication, and thereby, give the complete dose, can also be difficult to manage in a busy ward or outpatient unit, where a single nurse is managing several patients. The choice of administration line can also have consequences here. So-called *short sets* can allow independent intravenous channel rate-control over cytotoxic infusions to run into primary infusions of maintenance or hydration fluids. Administration lines with self-sealing connections that allow the clinician to unlock from a primary bag, lock to the medication to be given, complete the infusion, and run the medication bag dry while avoiding entry of any air into the administration line before switching back to the primary line can help with total delivery, the clinician needs to be aware of them and to know when to use them. Again, a bedside guide that can be consulted prior to medication administration preparation that identifies the material, light protection and filtration requirements, and the most effective administration line would add value.

This formative study was a step toward such a tool. We assayed the extent of the issue of lack of knowledge and information available to nursing staff and compounding pharmacists at the point of care. The survey method would need further development to be able to address pre- and postintervention education, with the addition of nonparametric testing for groups exposed to bedside tools and those who use traditional methods to choosing intravenous administration lines for specific medications.

## Appendix

Multimedia Appendix 1 Questionnaires.

Multimedia Appendix 2 Questionnaire answers.
