# Supplementary material for: Critical Care Nurses’ Knowledge of Correct Line Types for Administration of Common Intravenous Medications: Assessment and Intervention Study
Source: JMIR Form Res. 2022 Apr 26;6(4):e36710. doi: 10.2196/36710 (PMC9092237; doi:10.2196/36710)
Supplement: Multimedia Appendix 1 [file formative_v6i4e36710_app1.docx]

**Appendix One: Questionnaires for Critical Care and Oncology nursing staff administered via email survey, Google Forms response, and face-to-face.**

Dear respondent,

Thank you so much for helping us gather information on the current level of information and understanding of specialty IV-medication administration requirements among expert nurses.

As clinicians and educators, we are interested in how to serve you better with materials, guidance and support for clinical practice improvement in administering specialty infusions.

Please give only your own assessment for each question. Please do not use reference materials or ask colleagues, we hope to get a true ‘snapshot’ of the knowledge that specialist nurses take to the bedside every day when dealing with complex infusion therapy.

Your answers will help us in creating knowledge-strategies to support you in your everyday work.

Your answers are completely anonymous, confidential and will not be used in any commercial process.

Which speciality do you work within?

HDU □

ICU □

NICU □

PICU □

1. Which of the following medications would you use a light-protective IV set for?

(choose all that apply)

a. Adrenaline/Epinephrine b. Dobutamine

c. Midazolam d. Amiodarone

e. Labetalol f. Alteplase

g. Fentanyl h. Propofol

i. Nitroglycerin j. Digoxin

2. Which of the following medications would you use a Low-sorbing IV set for?

(choose all that apply)

a. Amiodarone b. Insulin

c. Heparin d. Midazolam

e. Nitroglycerin f. Alteplase

g. Omeprazole h. Levofloxacin

i. Remifentanil j. Teicoplanin

3. Which of the following medications would you administer through a 0.2-micron filter?* (choose all that apply)

a. Amiodarone b. Alteplase (t-PA)

c. Dopamine d. Fentanyl

**Removed from final results.*

4. When administering TPN containing Lipids, which size filter should be used?

(choose all that apply)

a. 0.2-micron b. 1.2-micron

c. 15-micron d. 200-micron

5. For an infusion of Albumin 5%, which size filter would be required?

(choose all that apply)

a. 15-micron b. No filter

c. 1.2-micron d. 200-micron

6. Which type of set does an infusion of Epoprostenol require?

(choose all that apply)

a. Light-protective b. Low-sorbing

c. Filter d. No special requirements required
