# Supplementary material for: Critical Care Nurses’ Knowledge of Correct Line Types for Administration of Common Intravenous Medications: Assessment and Intervention Study
Source: JMIR Form Res. 2022 Apr 26;6(4):e36710. doi: 10.2196/36710 (PMC9092237; doi:10.2196/36710)
Supplement: Multimedia Appendix 2 [file formative_v6i4e36710_app2.docx]

**Appendix Two: Survey Correct Answers with supporting references.**

| 1. | 2. | 4. | 5. | 6. |
| --- | --- | --- | --- | --- |
| Which of the following medications would you use a light-protective IV set for? (choose all that apply) | Which of the following medications would you use a Low-sorbing IV set for? (choose all that apply) | When administering TPN containing Lipids, which size filter should be used? (choose all that apply) | For an infusion of Albumin 5%, which size filter would be required? (choose all that apply) | Which type of set does an infusion of Epoprostenol require? (choose all that apply) |
| Adrenaline/Epinephrine [A1-A5]  Amiodarone [A5-A11]  Digoxin [A5, A6, A12-A13]  Labetalol [A5-A6, A23-A24] | Amiodarone [A5-A11]  Insulin [A15-17]  Nitroglycerin [A5-A6, A11, A18-A20] | 1.2 micron [A25] | No Filter [A26] | Light Protection [A27]  Filter [A28] |

**Survey Correct Answers References:**

A1. <https://www.medicines.org.uk/emc/product/3447/smpc#gref>

Accessed October 2020.

A2. <https://www.rxlist.com/adrenalin-drug.htm#description>

Accessed October 2020.

A3. FDA Label Epinephrine 2016 via <https://www.accessdata.fda.gov/drugsatfda_docs/label/2016/205029s002lbl.pdf>

Accessed February 2021.

A4. Stabilis Monographie Epinephrine hydrochloride Accessed 22nd February 2021 via <https://www.stabilis.org/Monographie.pdf.php?Molecule=Epinephrine%20hydrochloride>

A5. Tønnesen, Hanne Hjorth. Medicines and light- ensuring patient safety: Photostability of parenteral products. University of Oslo, Department of Pharmacy. 2020. 07 Media. 44-46.

A6. University of Illinois at Chicago College of Pharmacy, Drug Information Group* (2014). Light-protective injectable prescription drugs. Hospital pharmacy, 49(2), 136–163. <https://www.ncbi.nlm.nih.gov/pmc/articles/PMC3940680/>

A7. Sanofi. Cordarone x Data Sheet 31 October 2018. via <https://www.sanofi.com.au/-/media/Project/One-Sanofi-Web/Websites/Asia-Pacific/Sanofi-AU/en/Home/Our-Products/Prescription-Medicines/New-Zealand/docs/cordarone-x-ds.pdf?la=en&hash=A4CD8EE26DB57C55F0E92F1E64791958>

Accessed February 2021.

A8. Stabilis Monographie Amiodarone hydrochloride via <https://www.stabilis.org/Monographie.pdf.php?Molecule=Amiodarone%20hydrochloride>

Accessed October 2020.

A9. Moser R, Becker J. To Filter or not to filter? Lehigh Valley Health Network LVHN Scholarly Works Patient Care Services / Nursing. Accessed 23rd February 2021 via <https://scholarlyworks.lvhn.org/cgi/viewcontent.cgi?article=1344&context=patient-care-services-nursing#:~:text=An%20increased%20risk%20of%20phlebitis,necessary%20component%20when%20administering%20amiodarone>.

A10. Nexterone® Label information. via <https://www.accessdata.fda.gov/drugsatfda_docs/label/2010/022325s001lbl.pdf>

Accessed October 2020.

A11. Maraiki F, Farooq F, Ahmed M, Eliminating the use of intravenous glass bottles using a FOCUS-PDCA model and providing a practical stability reference guide, International Journal of Pharmacy Practice 2016, 24, pp. 271-282. <https://onlinelibrary.wiley.com/doi/epdf/10.1111/ijpp.12245>

A12. Riley CM. Stability of milrinone and digoxin, furosemide, procainamide hydrochloride, propranolol hydrochloride, quinidine gluconate, or verapamil hydrochloride in 5% dextrose injection. Am J Hosp Pharm ; 45: 2079-2091. 1988.

A13. Stabilis Monographie Digoxin. via <https://www.stabilis.org/Monographie.pdf.php?Molecule=Digoxin>

Accessed October 2020.

A14. FDA Label for Lanoxin 20th September 2018. via <https://www.accessdata.fda.gov/drugsatfda_docs/label/2018/009330s033lbl.pdf>

Accessed October 2020.

A15. Thompson CD, Vital-Carona J, Faustino EV. The effect of tubing dwell time on insulin adsorption during intravenous insulin infusions. Diabetes Technology & Therapeutics. 2012 Oct;14(10):912-916. DOI: 10.1089/dia.2012.0098.

A16. FDA Label Humulin Reference ID:4517150, revised 11/2019. via <https://www.accessdata.fda.gov/drugsatfda_docs/label/2019/018780s175s176lbl.pdf>

Accessed October 2020.

A17. Stabilis Monographie Insulin. via <https://www.stabilis.org/Monographie.pdf.php?Molecule=Insulin>

Accessed February 2021.

A18. Treleano A, Wolz G, Brandsch R, Welle F. Investigation into the sorption of nitroglycerin and diazepam into PVC tubes and alternative tube materials during application, December 2008. International Journal of Pharmaceutics 369(1-2):30-7

A19. Stabilis Monographie Nitroglycerin. via <https://www.stabilis.org/Monographie.pdf.php?Molecule=Nitroglycerin>

Accessed February 2021.

A20. FDA Label, Nitroglycerin, Baxter Healthcare Corporation. via <https://www.accessdata.fda.gov/drugsatfda_docs/label/2014/019970s015lbl.pdf>

Accessed February 2021.

A21. Stabilis Monographie Midazolam Hydrochloride. via <https://www.stabilis.org/Monographie.pdf.php?Molecule=Midazolam%20hydrochloride>

Accessed October 2020.

A22. FDA Label Midazolam Fresenius Kabi. via <https://www.accessdata.fda.gov/drugsatfda_docs/label/2017/208878Orig1s000lbl.pdf>

Accessed October 2020.

A23. Stabilis Monographie Labetalol hydrochloride. via <https://www.stabilis.org/Monographie.pdf.php?Molecule=Labetalol%20hydrochloride>

Accessed February 2021.

A24. FDA label Labetalol Hydrochloride, Hospira. via <https://www.accessdata.fda.gov/drugsatfda_docs/label/2012/075239s010lbl.pdf>

Accessed February 2021.

A25. Rees Doyle, G & McCutcheon, J. Clinical Procedures for Safer Patient Care. Chapter 8 Intravenous Therapy; 8.8 Total Parenteral Nutrition (TPN). BCcampus Open Education. May 2021. <https://opentextbc.ca/clinicalskills/chapter/8-8/>

A26. FDA Label Albumin 5% Accessed via

<https://www.fda.gov/media/70406/download>

Accessed February 2021.

A27. <https://www.medicines.org.uk/emc/product/5291/smpc#gref>

Accessed February 2021.

A28.<https://gskpro.com/content/dam/global/hcpportal/en_US/Prescribing_Information/Flolan/pdf/FLOLAN-PI-PIL.PDF>

Accessed February 2021.
